# Impact of frailty on post-procedural adverse outcomes in older adults aged ≥75 years undergoing percutaneous coronary intervention: a systematic review of observational studies

**DOI:** 10.3389/fmed.2026.1819203

**Published:** 2026-05-14

**Authors:** Peng Tian, Liping Wang, Tianjiao He, Qiurong Zhang, Yingying Cai

**Affiliations:** 1 Department of Geriatrics, The First Affiliated Hospital of Chengdu Medical College, Chengdu, Sichuan, China; 2 School of Clinical Medicine, Chengdu Medical College, Chengdu, Sichuan, China; 3 Sichuan Geriatrics Clinical Medical Research Center, Chengdu, Sichuan, China

**Keywords:** complications, elderly patients, frailty, mortality, percutaneous coronary intervention, systematic review

## Abstract

**Purpose:**

The global aging population has led to a growing number of elderly patients aged ≥75 years undergoing percutaneous coronary intervention (PCI). Frailty is a common geriatric syndrome in this population, related to decreased physiological reserve and reduced ability to cope with stress, and it may significantly affect surgical outcomes. This systematic review aims to comprehensively evaluate the impact of frailty on major clinical outcomes (such as in-hospital mortality, all-cause mortality, bleeding, and stroke) in patients aged ≥75 years after percutaneous coronary intervention.

**Methods:**

We systematically searched PubMed, Embase, Web of Science, and the Cochrane Library from inception to December 2025. We included retrospective or prospective cohort studies reporting the relationship between frailty (assessed by any validated tool) and prognosis in elderly patients aged ≥75 years undergoing PCI. Data extraction covered study characteristics, population, age, CAD type, frailty assessment, and outcomes. The Newcastle–Ottawa Scale assessed bias risk. A fixed-effect model was used if *I*^2^ < 50%; otherwise a random-effects model was used. Subgroup, meta-regression analysis and sensitivity analyses explored heterogeneity sources. Funnel plots assessed publication bias. The GRADE system rated evidence quality for each outcome.

**Results:**

This systematic review included a total of 18 cohort studies, comprising 11 retrospective and 7 prospective studies, involving 2,038,546 patients. Our pooled analysis demonstrated that, compared with their non-frail counterparts, frail patients aged ≥75 years had a significantly higher risk of mortality and adverse outcomes following PCI. Specifically, frailty was closely associated with an increased risk of in-hospital death (RR = 3.16, 95% CI: 1.28–7.78) and all-cause death (RR = 2.51, 95% CI: 1.78–3.56). Furthermore, frailty significantly increased the incidence of complications, including the risk of bleeding (RR = 2.26, 95% CI: 1.54–3.31) and stroke (RR = 1.90, 95% CI: 1.86–1.94). The GRADE evidence certainty was low across all outcomes.

**Conclusion:**

In patients aged ≥75 years undergoing PCI, frailty is associated with increased risks of in-hospital mortality, all-cause mortality, bleeding, and stroke. These findings support perioperative risk stratification, shared decision-making, and individualized management, though low GRADE evidence and uncertainty warrant caution.

## Introduction

The global population is undergoing a profound and irreversible demographic shift toward aging. As the proportion of the elderly population continues to rise, the prevalence and associated disease burden of cardiovascular disease, particularly coronary heart disease (CHD), are increasing significantly, establishing it as one of the most critical health threats (1). Reflecting this demographic trend, the age profile of patients undergoing percutaneous coronary intervention (PCI), a cornerstone of coronary revascularization, is also shifting toward an older population (2). In this context, older patients, particularly those aged ≥75 years, have gradually become a significant population undergoing coronary stent implantation (3).

Patients aged ≥75 years undergoing PCI exhibit a distinct clinical phenotype, characterized by a high prevalence of clustered cardiometabolic risk factors, age-related physiological decline across multiple organ systems, and the common challenge of polypharmacy (4). This population has a significantly reduced physiological tolerance to the stress of surgery and to periprocedural bleeding and ischemic events, which greatly complicates the treatment decision-making process (5). The core challenge in clinical practice lies in precisely weighing the potential benefits of revascularization against its inherent risks, in order to strike a delicate balance between aggressive lesion intervention and the avoidance of overtreatment.

Frailty is a clinical syndrome characterized by a decline in physiological reserve resulting from the deterioration of multiple physiological systems, with its core feature being a significantly increased vulnerability to stressors (6). Epidemiological data indicate that the prevalence of frailty among community-dwelling older adults aged 65 years and older is approximately 12.2%, a proportion that rises sharply to 33.2% in those aged ≥75 years (7). Importantly, frailty is no longer viewed merely as an inevitable concomitant of the aging process, but is now widely recognized as an independent risk factor that strongly and significantly predicts adverse clinical outcomes and differential responses to treatment.

Although previous studies have established frailty as a powerful predictor of adverse outcomes in older adults with cardiovascular disease-significantly associated with increased risks of mortality, rehospitalization, bleeding events, and functional decline (8)—existing systematic reviews and meta-analyses have important limitations. Many have included patients across all ages or broadly defined “older” PCI populations (8), while those specifically focusing on patients aged ≥75 years have been inconclusive due to small sample sizes and a lack of comparative evidence (9). In the past 2 years, several large-sample studies (10, 11) have been published, potentially providing new and robust evidence. This study aims to conduct a systematic review and meta-analysis to systematically integrate evidence from existing cohort studies, with the objective of quantitatively assessing the impact of pre-procedural frailty on short- and long-term prognosis in patients aged ≥75 years undergoing coronary stent implantation.

## Material and methods

### Study design and reporting guidelines

This study is a systematic review and meta-analysis aimed at evaluating the impact of frailty status on clinical outcomes after PCI in elderly patients aged ≥75 years. This review was conducted and reported in accordance with the preferred reporting items for systematic reviews and meta-analyses (PRISMA) 2020 statement to ensure transparency and reproducibility of the methodology.

### Inclusion and exclusion criteria

The following inclusion criteria were applied to select studies for this review: (1) Studies only include retrospective cohort studies or prospective cohort studies; (2) Patients aged ≥75 years who underwent elective or emergency percutaneous coronary intervention with stent implantation for any clinical indication (stable coronary artery disease or acute coronary syndrome); (3) Patients pre-proceduraly assessed as “frail” using any validated objective frailty assessment tool (such as the Fried Frailty Phenotype, Clinical Frailty Scale, Frailty Index, FRAIL scale, etc.); (4) Age-matched patients undergoing surgery during the same period who were pre-proceduraly assessed by the same or similar tool and determined to be “non-frail” (such as robust or pre-frail); (5) Containing primary outcome measures: in-hospital mortality, all-cause mortality (follow-up ≥30 days). Major bleeding events (according to study definition or Bleeding Academic Research Consortium (BARC) ≥ grade 2); stroke (including ischemic or hemorrhagic stroke). The exclusion criteria were: (1) Studies that did not use an objective, validated tool to assess frailty; (2) Studies that did not provide comparable data on outcome measures between the frail and non-frail patient groups (e.g., studies that only provided data for a single group); (3) Case reports, commentaries, reviews, editorials, conference abstracts (unless full data could be obtained), and systematic reviews; (4) Non-Chinese and non-English language publications; (5) Duplicate publications or studies with overlapping data (the study with the most complete or most recent data was retained).

### Search strategy

We systematically searched PubMed, Embase, the Cochrane Library, and Web of Science. The search timeframe was set from the inception of each database to December 2025. The search strategy used a combination of subject headings and free-text terms, and was adjusted according to the syntax rules of each database. The main search strategy included: “frailty,” “elderly,” “percutaneous coronary intervention/stent implantation,” and “prognosis.” Additionally, we manually checked the reference lists of the included studies to supplement any potentially missed literature. The search strategy employed for PubMed is detailed below:

#1 “Percutaneous Coronary Intervention”[Mesh] OR “Coronary Artery Disease/surgery”[Mesh] OR “Drug-Eluting Stents”[Mesh] OR (PCI[tiab] OR “percutaneous coronary”[tiab] OR “coronary stent^*^”[tiab] OR “drug-eluting stent^*^”[tiab])

#2 “Frailty”[Mesh] OR “Geriatric Assessment”[Mesh] OR (frail^*^[tiab] OR fragil^*^[tiab] OR “clinical frailty scale”[tiab] OR “fried frailty”[tiab] OR “cumulative deficit”[tiab])

#3 “Aged”[Mesh] OR “Aged, 80 and over”[Mesh] OR (aged[tiab] OR elderly[tiab] OR geriatric[tiab] OR “older patient^*^”[tiab] OR octogenarian^*^[tiab] OR nonagenarian^*^[tiab])

#4 (age[tiab] AND (“75”[tiab] OR “75”[tiab] OR “80”[tiab] OR “85”[tiab] OR “90”[tiab])) OR “seventy five”[tiab] OR “eighty”[tiab]

#5 #1 AND #2 AND (#3 OR #4)

#6 controlled clinical trial[pt] OR observational study[pt] OR cohort[tiab] OR prospective[tiab] OR retrospective[tiab]) NOT (animals[mh] NOT humans[mh])

#7 #5 AND #6

### Data extraction

Two reviewers independently performed the literature screening and data extraction. After removing duplicates using reference management software, two reviewers independently screened titles and abstracts to identify potentially eligible studies. The full texts of these studies were then independently assessed against the pre-specified inclusion criteria. Any disagreements between the reviewers were resolved through discussion or, if necessary, by consultation with a third reviewer. Since all included studies were observational, we extracted adjusted risk estimates (e.g., multivariate-adjusted odds ratios or risk ratios) reported by each study to control for confounding bias. A standardized data extraction form was designed. The extracted content included: basic study information; patient characteristics; intervention details (type of surgery); frailty assessment tools; and outcome measures.

### Quality assessment

The Newcastle–Ottawa Scale was used to evaluate the methodological quality of the included cohort studies. The NOS scale scores studies across three domains: selection of study populations (4 points), comparability of groups (2 points), and outcome measurement (3 points), for a total of 9 points. Studies with a score of ≥7 are generally considered to be high-quality studies. The assessment process was completed independently by two researchers.

### Statistical analysis

If studies had sufficient clinical and methodological homogeneity in terms of population characteristics, type of procedure, frailty definition, and outcome measurement, a quantitative synthesis was performed. We calculated the relative risks and their corresponding 95% confidence intervals for in-hospital mortality, all-cause mortality, bleeding, and stroke. Heterogeneity among the included studies was assessed using the *I*^2^ statistic: when the *I*^2^ value was ≤ 50%, indicating low heterogeneity, we used a fixed-effect model in the meta-analysis; when the *I*^2^ value was >50%, a random-effects model was used. To explore potential sources of significant heterogeneity (*I*^2^ > 50%), we first performed pre-specified subgroup analyses and then conducted meta-regression analyses. In addition, sensitivity analyses were performed by sequentially omitting each individual study to assess the impact of a single study on the overall pooled effect size. For outcomes reported by at least 10 studies, funnel plot asymmetry was assessed by visual inspection combined with Egger's test and Begg's test to evaluate the presence of potential publication bias. The (Grading of Recommendations Assessment, Development, and Evaluation, GRADE) system was used to rate the quality of evidence for each outcome measure. Observational studies start as low-quality evidence and can be downgraded based on risk of bias, inconsistency, indirectness, imprecision, and publication bias, or upgraded based on large effect size, dose-response gradient, and negative bias. The final quality of evidence is classified into four levels: high, moderate, low, and very low. All statistical analyses were performed using Stata version 15.0 or RevMan version 5.4 software, and a *P*-value < 0.05 was considered statistically significant.

## Results

### Study screening

**Figure 1:**
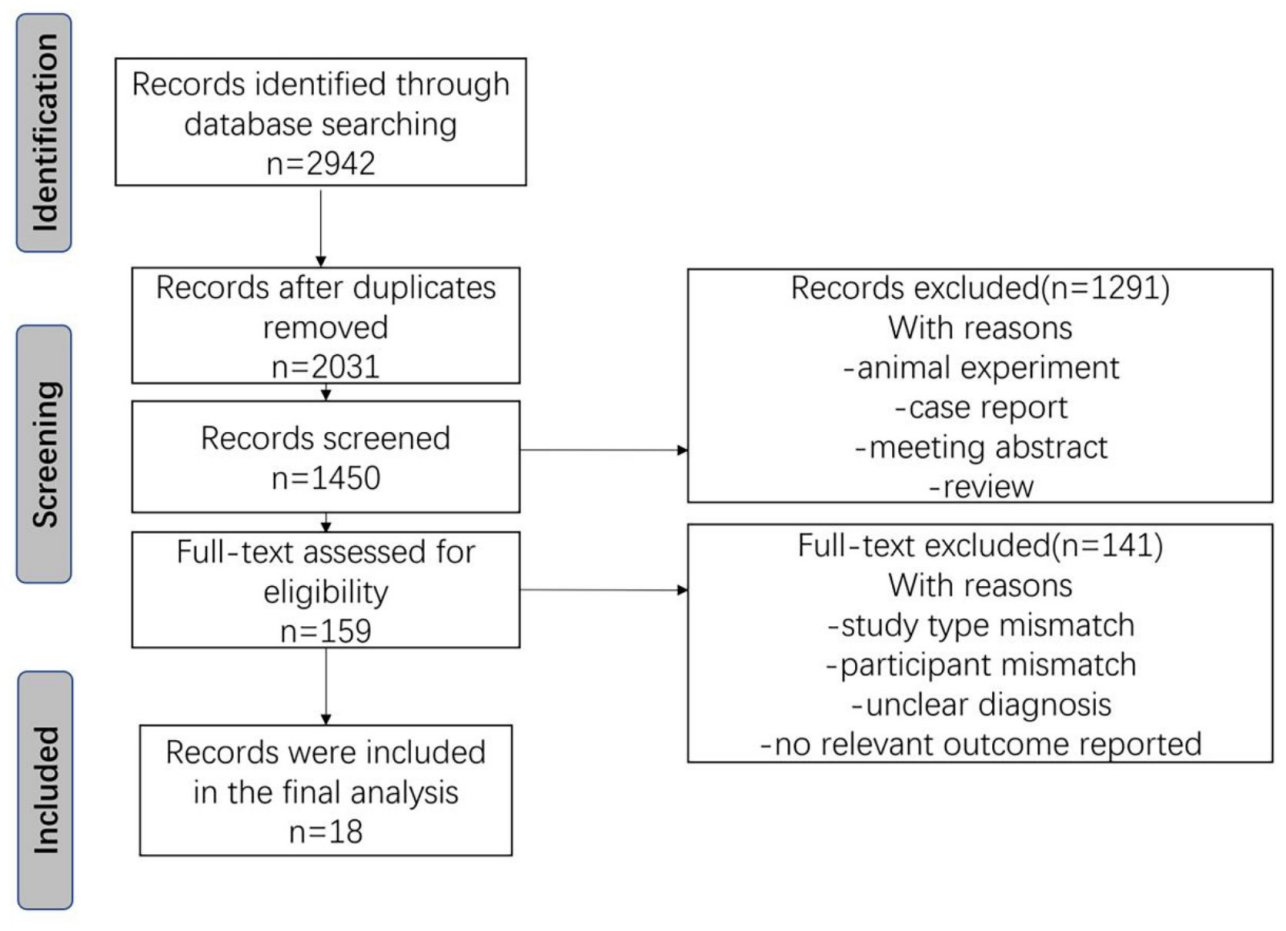
PRISMA flow chart of study screening.

A total of 2,942 records were retrieved through the initial search. Following the systematic screening of titles, abstracts, and full texts, 18 studies met the inclusion criteria and were included in the final review (Figure 1).

### Study characteristics

This systematic review included a total of 18 cohort studies from 6 countries (including 7 prospective studies and 11 retrospective studies), involving 2,038,546 patients. As shown in Table 1, all 18 studies were rated as high quality. The characteristics of the nine different frailty scales in Table 2.

**Table 1:** Characteristics of the included studies.

Author	Year	Study design	Location	Age	ACS subcalss	Frailty scale	Sample	Surgical approach	Outcome measures
Liao et al. (15)	2018	Prospective cohort study	Spain	86.7 (4.0)	NSTEACS	FRAIL Scale	145	Invasive strategy	In-hospital mortality
Dodson et al. (21)	2018	Prospective cohort study	U.S	83.6 (3.8)	AMI (STEMI/NSTEMI)	Study-specific measure	3,311	Coronary angiography	In-hospital major bleeding
Damluji et al. (22)	2019	Retrospective cohort study	U.S	85.9	AMI	Cumulative frailty index (CFI)	13,832	PCI	In-hospital mortality
Kwok et al. (23)	2019	Retrospective cohort study	U.S	80.0 (11)	ACS	Hospital frailty risk score (HFRS)	99,6901	PCI	In-hospital mortality, in-hospital major bleeding
Nishihira et al. (24)	2020	Prospective Cohort study	Japan	92 (91–94)	AMI	Fried phenotype scoring system (FPSS)	96	PCI	In-hospital major bleeding, in-hospital mortality, all-cause mortality
Yoshioka et al. (25)	2019	Retrospective cohort study	Japan	84.6 ± 3.8	STEMI	Clinical frailty scale (CFS)	273	PCI	In-hospital mortality, all-cause mortality
Hermans et al. (26)	2019	Retrospective cohort study	Netherlands	79 ± 6.4	STEMI	VMS score	206	PCI	All-cause mortality
Nishihira et al. (27)	2021	Prospective cohort study	Japan	84.5 (82–88)	AMI	FPSS	546	PCI	All-cause mortality, in-hospital major bleeding
Calvo et al. (28)	2019	Prospective cohort study	Spain	82.6 ± 6	STEMI	Frailty score (FS)	259	PCI	In-hospital mortality, all-cause mortality
Sujino et al. (29)	2015	Retrospective cohort study	Japan	88.1	STEMI	CFS	42	PCI	In-hospital mortality
Wong et al. (30)	2019	Retrospective cohort study	Netherlands	87.6 ± 2.8	ACS	Edmonton frail scale (EFT)	180	PCI	All-cause mortality
Roman et al. (10)	2024	Retrospective cohort study	England	81.0 (72.0–87.0)	ACS	HFRS	565,378	PCI/CABG	One-year mortality, five-year mortality, stroke, major bleeding
Tashiro et al. (31)	2022	Retrospective cohort study	Japan	84.4 (3.7)	STEMI	CFS	244	PCI	In-hospital events (\Stroke)
ÖZBEK et al. (32)	2023	Retrospective cohort study	Turkey	84.6 ± 3.4	ACS	CFS	113	PCI	Major bleeding, all-cause mortality
Batty et al. (33)	2018	Prospective cohort study	UK	81 ± 4	NSTEACS	FRAIL-HEART	77	PCI/CABG	All-cause mortality, stroke, in-hospital major bleeding
Ratcovich et al. (34)	2022	Prospective cohort study	UK	81.2 (4.1)	NSTEACS	CFS	185	PCI	All-cause mortality, stroke, in-hospital major bleeding
Gallo et al. (35)	2025	Retrospective cohort study	Spain	80 (78–84)	(LM) disease	FRAIL Scale	68	PCI	All-cause mortality, stroke
Popat et al. (11)	2025	Retrospective cohort study	U.S	≥75	NSTEMI	HFRS	456,690	PCI	In-hospital mortality, In-hospital major bleeding, stroke

**Table 2:** Characteristics of the frailty scales.

Classification	Core role	Typical characteristics	Frailty scale
Screening tools	Quickly identify at-risk groups	Few items, short duration, easy to operate	FRAIL scale, groningen frailty indicator (GFI), clinical frailty scale (CFS)
Comprehensive assessment tools	Multidimensional precise assessment and definition	Detailed assessment, provision of rich information, commonly used in scientific research	Fried phenotype (FPSS), edmonton frailty scale (EFS), cumulative frailty index (CFI), FRAIL-HEART
Risk stratification tools	Using administrative data to predict hospitalization risk	ithout direct patient contact, based on coded data, for predictive purposes	Hospital frailty risk score (HFRS)
Study-specific tool	Serve specific research or institutions	Limited generalizability, varied forms	VMS score, study-specific measure, frailty score (FS)

## Mortality

### In-hospital mortality

**Figure 2:**
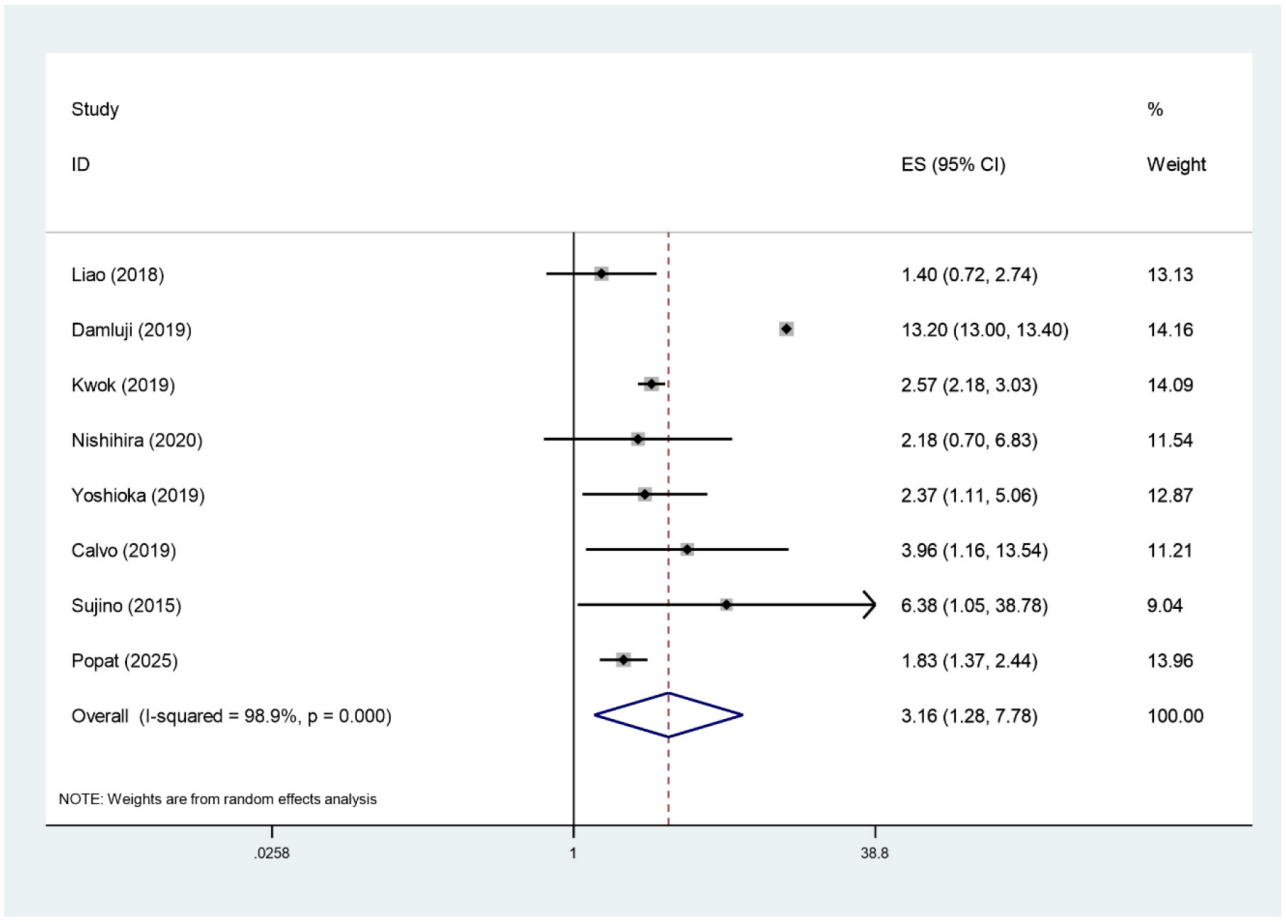
Forest plot of in-hospital mortality rates in frail patients.

A total of eight studies, comprising 1,468,238 patients, examined the association between pre-procedural frailty and in-hospital mortality following PCI in patients aged ≥75 years. The meta-analysis results showed high heterogeneity (*I*^2^ = 98.9%), so we used a random-effects model for the analysis. The results showed that frailty was significantly associated with in-hospital mortality in patients aged ≥75 years (RR = 3.16, 95% CI: 1.28–7.78), as shown in Figure 2. After excluding the Damluji study: (*I*^2^ = 26.6%), (RR = 2.34, 95% CI: 2.04, 2.68); after excluding the Kwok study: (*I*^2^ = 97.7%), (RR = 3.27, 95% CI: 1.12, 9.51); after excluding the Popat study: (*I*^2^ = 98.7%), (RR = 3.45, 95% CI: 1.35, 8.80). All differences were statistically significant.

### All-cause mortality

**Figure 3:**
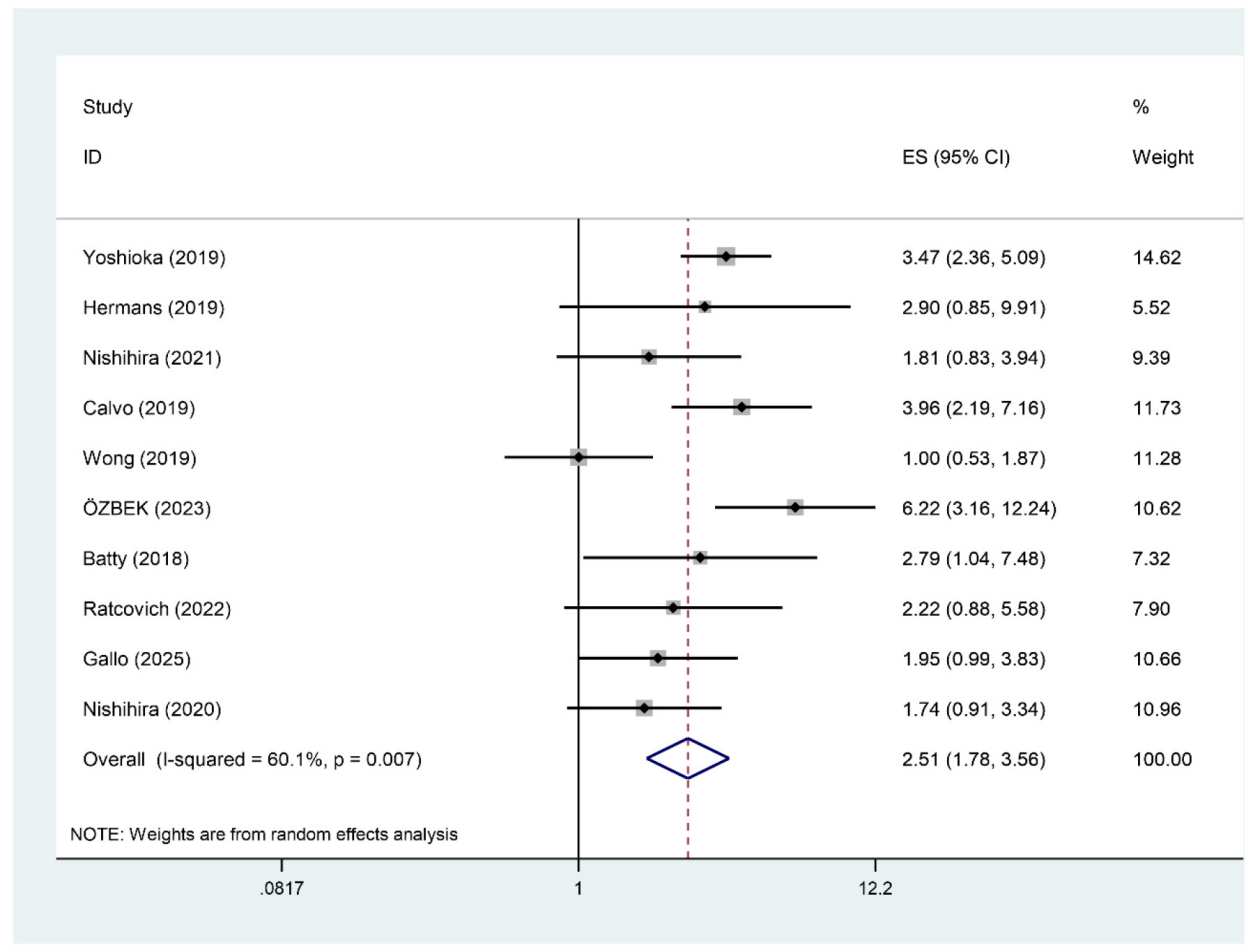
Forest plot of all-cause mortality rates in frail patients.

Ten studies, encompassing 2,003 patients, examined the association between pre-procedural frailty and all-cause mortality following PCI in patients aged ≥75 years. The meta-analysis results showed moderate heterogeneity (*I*^2^ = 60.1%), so a random-effects model was used for the analysis. The results showed that frailty was significantly associated with an increased risk of all-cause mortality in patients aged ≥75 years (RR = 2.51, 95% CI: 1.78–3.56), as shown in Figure 3.

### Overall complications

#### Major Bleeding

**Figure 4:**
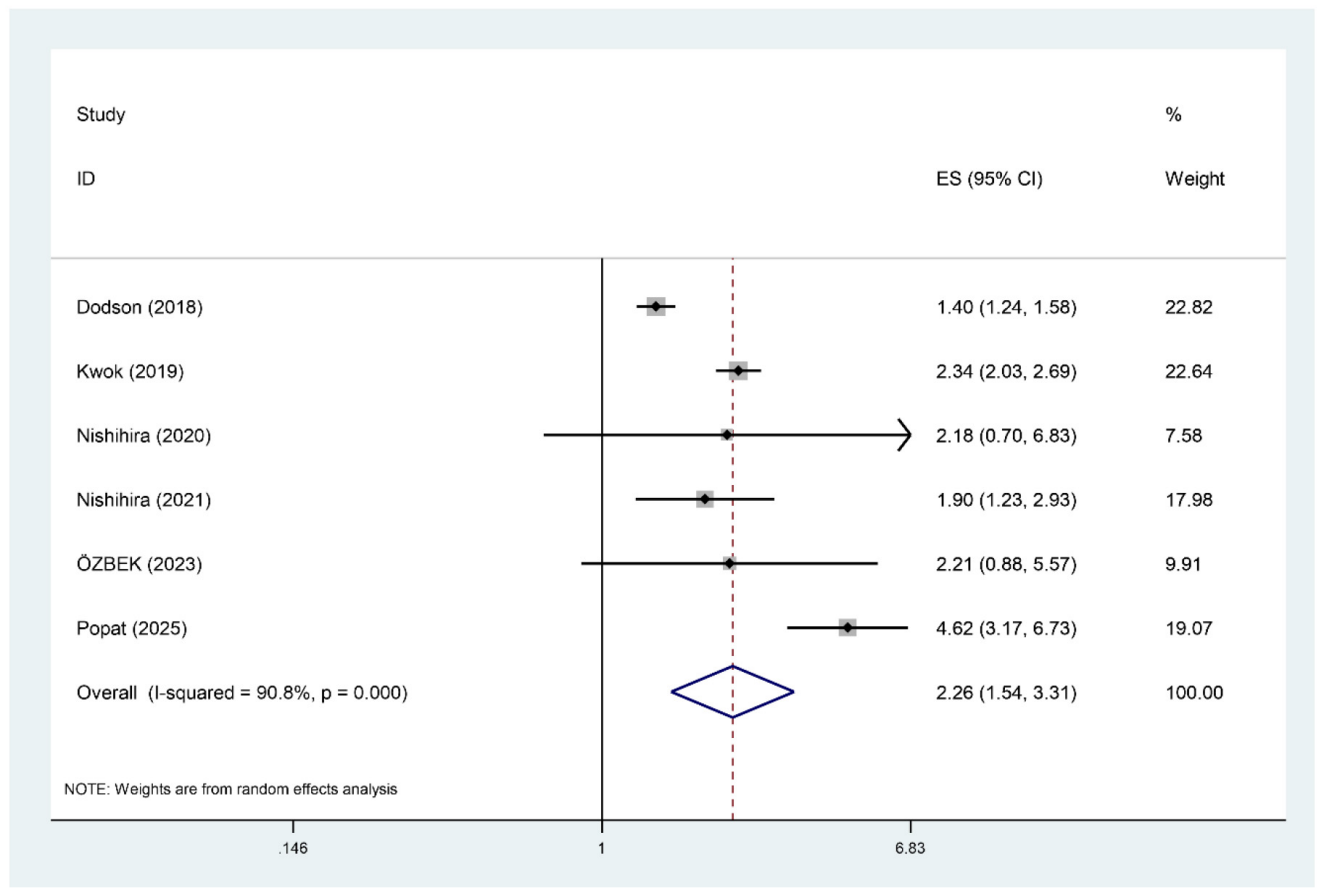
Forest plot of major bleeding in frail patients.

Six studies, comprising 1,457,657 patients, assessed the association between pre-procedural frailty and bleeding events following PCI in patients aged ≥75 years. The meta-analysis results showed high heterogeneity (*I*^2^ = 90.8%), so a random-effects model was used for the analysis. The results showed that frailty was significantly associated with an increased risk of bleeding in patients aged ≥75 years (RR = 2.26, 95% CI: 1.54–3.31), as shown in Figure 4. After excluding the Kwok study: (*I*^2^ = 89.1%), (RR = 2.25, 95% CI: 1.26, 4.02); after excluding the Popat study: (*I*^2^ = 86.6%), (RR = 1.89, 95% CI: 1.33, 2.68). All differences were statistically significant.

#### Stroke

**Figure 5:**
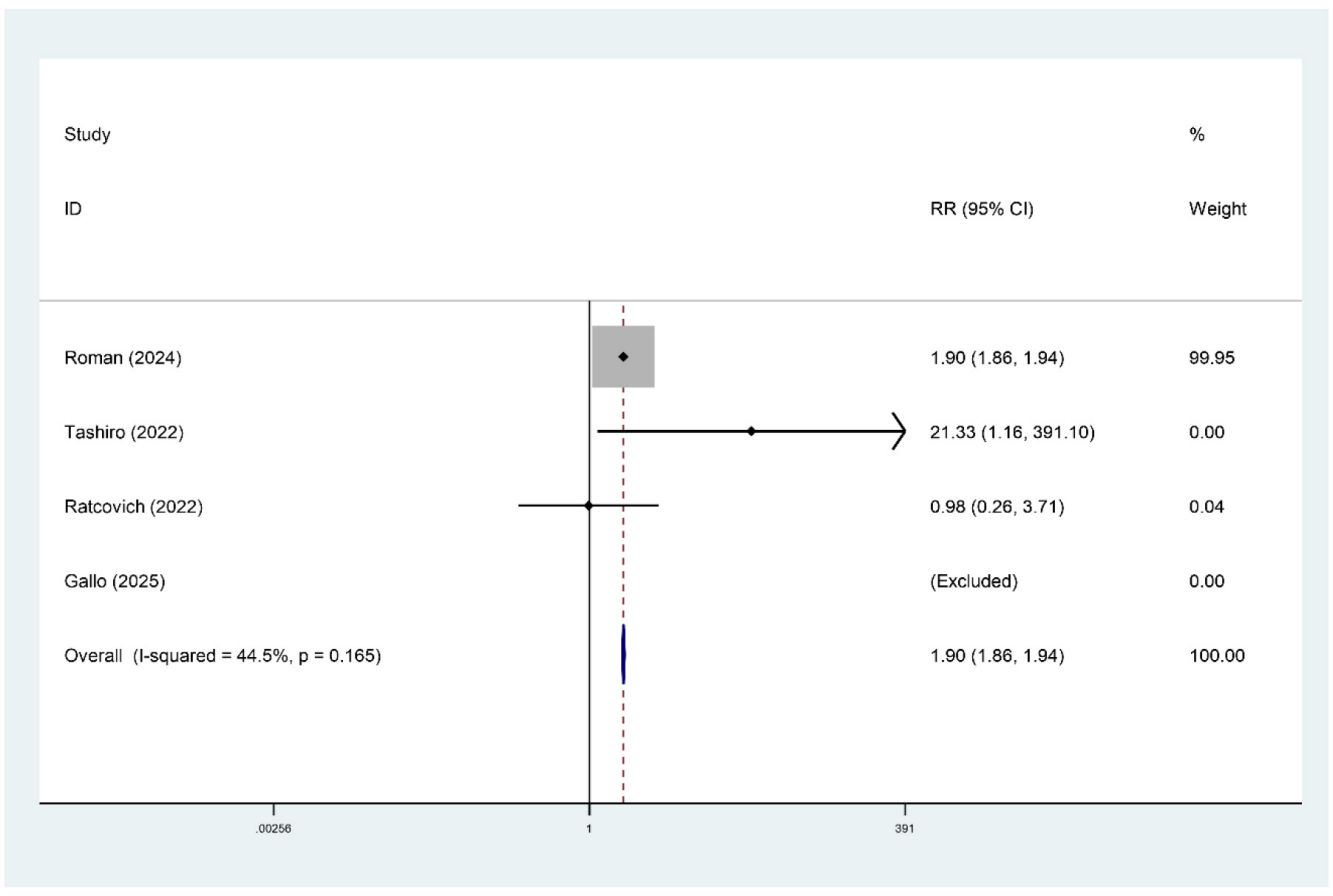
Forest plot of stroke in frail patients.

Four studies, comprising 565,875 patients, assessed the association between pre-procedural frailty and stroke following PCI in patients aged ≥75 years. The meta-analysis results showed mild heterogeneity (*I*^2^ = 44.5%), so a fixed-effect model was used for the analysis. The results showed that frailty was significantly associated with an increased risk of stroke in patients aged ≥75 years (RR = 1.90, 95% CI: 1.86–1.94), as shown in Figure 5. After excluding the Roman study, the meta-analysis showed high heterogeneity (*I*^2^ = 74%), and the difference between the two groups was not statistically significant (RR = 3.52, 95% CI: 0.16, 77.20).

## Sensitivity analysis, subgroup analysis and meta-regression

Sensitivity analysis did not find the source of heterogeneity. Meta-regression analysis showed that myocardial infarction classification (Non-ST-Segment Elevation Myocardial Infarction, NSTEMI; ST-Segment Elevation Myocardial Infarction, STEMI) was the source of heterogeneity for in-hospital mortality (*P* = 0.00) and bleeding events (*P* = 0.04). Subgroup analysis showed that there was low heterogeneity in the results of prospective studies. Screening scale type showed low heterogeneity in predicting in-hospital mortality, while comprehensive assessment tools showed low heterogeneity in predicting all-cause mortality and bleeding. Substantial geographic heterogeneity was observed for most outcomes when analyses were stratified by study location. The NSTEMI subgroup showed higher heterogeneity in all-cause mortality and bleeding events (Table 3).

**Table 3:** Subgroup analysis of frailty associated with PCI.

Main outcome measure	Numbers	RRs	CIs	*I* ^2^	Design type
In-hospital mortality	3	1.85	1.10, 3.13	9.8	Prospective
	5	3.83	1.25, 11.71	99.3	Retrospective
All-cause mortality	5	2.04	1.57, 2.65	0	Prospective
	4	2.73	1.24, 5.99	73.8	Retrospective
Major bleeding	3	1.44	1.28, 1.61	12.6	Prospective
	3	2.98	1.75, 5.08	82	Retrospective
Stroke	1	0.98	0.26, 3.71	Not applicable	Prospective
	3	4.04	0.45, 35.29	62.3	Retrospective
**Main outcome measure**	**Numbers**	**RRs**	**CIs**	*I* ^2^	**Types of frailty scales**
In-hospital mortality	3	1.93	1.19, 3.14	29.8	Rapid screening scale
	2	5.89	1.02, 34.09	89.5	Multidimensional assessment tool
	2	2.22	1.59, 3.08	75	Risk stratification tool
	1	3.96	1.15, 13.54	Not applicable	Study-specific tool
All-cause mortality	4	3.23	2.03, 5.13	53.7	Rapid screening scale
	4	1.55	1.08, 2.23	14.8	Multidimensional assessment tool
	Not applicable	Not applicable	Not applicable	Not applicable	Risk stratification tool
	2	3.73	2.19, 19.54	0	Study-specific tool
Major bleeding	1	2.21	0.88, 5.57	Not applicable	Rapid screening scale
	2	1.93	1.29, 2.90	0	Multidimensional assessment tool
	2	3.21	1.65, 6.25	90.9	Risk stratification tool
	1	1.4	1.24, 1.58	Not applicable	Study-specific tool
Stroke	3	3.52	0.16, 77.2	74	Rapid screening scale
	Not applicable	Not applicable	Not applicable	Not applicable	Multidimensional assessment tool
	1	1.9	1.86,1.94	Not applicable	Risk stratification tool
	Not applicable	Not applicable	Not applicable	Not applicable	Study-specific tool
**Main outcome measure**	**Numbers**	**RRs**	**CIs**	*I* ^2^	**Regions**
In-hospital mortality	2	2.06	0.77, 5.52	52.8	Europe
	3	3.98	1.00, 15.85	99.6	North America
	3	2.58	1.42, 4.68	0	Asia
	2	1.49	0.54, 4.07	56.3	Oceania
All-cause mortality	4	3.32	1.96, 5.62	55.5	Europe
	1	2.79	1.04, 7.48	Not applicable	North America
	3	2.4	1.45, 3.97	54.6	Asia
	2	1.49	0.54, 4.07	56.3	Oceania
Major bleeding	1	2.21	0.88, 5.57	Not applicable	Europe
	3	2.4	1.42, 4.04	95.3	North America
	2	1.93	1.29, 2.90	0	Asia
	Not applicable	Not applicable	Not applicable	Not applicable	Oceania
Stroke	3	1.9	1.86, 1.94	0	Europe
	Not applicable	Not applicable	Not applicable	Not applicable	North America
	1	21.33	1.16, 391.1	Not applicable	Asia
	Not applicable	Not applicable	Not applicable	Not applicable	Oceania
**Main outcome measure**	**Numbers**	**RRs**	**CIs**	*I* ^2^	**MI (NSTEMI/STEMI)**
In-hospital mortality	4	2.11	1.62, 2.76	51.9	NSTEMI
	4	5.64	1.99, 16.03	78.3	STEMI
All-cause mortality	7	2.15	1.36, 3.40	62.7	NSTEMI
	3	1.27	0.96, 1.58	0	STEMI
Major bleeding	3	2.98	1.75, 5.08	82	NSTEMI
	3	1.44	1.28, 1.61	12.6	STEMI
Stroke	3	1.9	1.86, 1.94	0	NSTEMI
	1	21.33	1.16, 391.10	Not applicable	STEMI

## Publication bias

**Figure 6:**
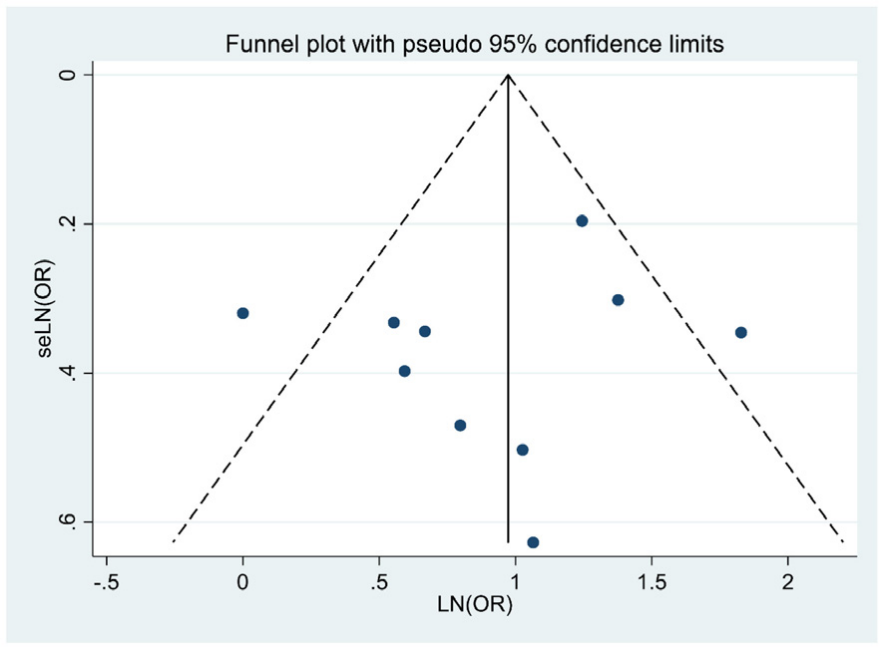
Funnel plot of all-cause mortality.

Publication bias was assessed for the outcome of all-cause mortality, for which more than 10 studies were available. The funnel plot was symmetric, as shown in Figure 6. Begg's test and Egger's test were used for quantitative analysis, and no potential publication bias was found.

## GRADE quality of evidence

The four outcome measures included in this study were all derived from observational studies, and according to the GRADE system rating, the quality of evidence was low for all outcomes, as shown in Table 4.

**Table 4:** GRADE quality of evidence for outcome measures.

Outcome	Number of studies	Study design	Risk of bias	Inconsistency	Indirectness	Imprecision	Publication bias	Upgrading factors	Quality of evidence	Importance
In-hospital mortality	8	Cohort studies	Not serious	Serious^a^ (*I*^2^ = 98.9%)	Not serious	Not serious^b^	None	Large effect size (RR = 3.16 > 2)	Low ⊕⊕⊖⊖	Critical
All-cause mortality	10	Cohort studies	Not serious	Serious (*I*^2^ = 60.1%)	Not serious	Not serious	None	Large effect size (RR = 2.51 > 2)	Low ⊕⊕⊖⊖	Critical
Bleeding	6	Cohort studies	Not serious	Serious (*I*^2^ = 90.8%)	Not serious	Not serious	None	Large effect size (RR = 2.26 > 2)	Low ⊕⊕⊖⊖	Critical
Stroke	4	Cohort studies	Not serious	Not serious^c^ (*I*^2^ = 44.5%)	Not serious	Not serious^d^	None	None (RR = 1.90 < 2)	Low ⊕⊕⊖⊖	Critical

^a^In-hospital mortality: original *I*^2^ = 98.9% (very high heterogeneity). However, after excluding the Damluji study, *I*^2^ dropped to 26.6%, and all sensitivity analyses showed consistent direction of effect (all RRs >2 and statistically significant). Therefore, downgraded by one level rather than two.

^b^In-hospital mortality: the 95% CI (1.28–7.78) is relatively wide, but given the very large total sample size (1.46 million) and the lower bound still >1, no downgrade for imprecision.

^c^Stroke: original *I*^2^ = 44.5% (mild heterogeneity), no downgrade. However, excluding the Roman study increased heterogeneity (*I*^2^ = 74%) and the result became non-significant (RR = 3.52, 95% CI: 0.16–77.20), indicating limited robustness. This does not meet GRADE downgrading criteria but warrants caution.

^d^Stroke: the 95% CI (1.86–1.94) is very narrow, indicating high precision.

## Discussion

This systematic review specifically focused on very elderly patients (≥75 years) undergoing percutaneous coronary intervention (PCI) and systematically evaluated the impact of pre-procedural frailty on major postprocedural clinical outcomes, incorporating data from 18 cohort studies comprising over 2 million patients. The results demonstrated that frailty is an independent and significant predictor of in-hospital mortality, long-term all-cause mortality, major bleeding, and stroke risk in very elderly patients undergoing PCI. Traditional risk scores (e.g., GRACE, SYNTAX) primarily integrate acute physiological and anatomical parameters (12, 13); incorporating frailty assessment can further identify “occult high-risk” patients-those classified as “intermediate risk” by traditional scores but with depleted physiological reserve-thereby enabling more refined risk stratification (14). These findings provide strong evidence to support the clinical integration of frailty assessment into periprocedural risk stratification for very elderly PCI patients, and also highlight the unique “bidirectional risk dilemma” faced by this population.

While a minority of studies (15) have reported null findings regarding the comparative effectiveness of conservative vs. invasive strategies in frail older adults, these were likely underpowered and susceptible to selection bias; conversely, the preponderance of evidence supports a significant association between frailty and adverse outcomes. This meta-analysis demonstrated that frailty conferred a >2-fold higher risk of in-hospital mortality (RR = 3.16, 95%CI: 1.28–7.78) and a 1.5-fold higher risk of all-cause mortality (RR = 2.51, 95%CI: 1.78–3.56) among very elderly PCI recipients. These findings are consistent with previous systematic reviews that included broader age ranges, but the effect sizes in our study were larger (16), suggesting that the prognostic value of frailty may be even more pronounced in the very old (≥75 years). As a core manifestation of geriatric syndromes, frailty-characterized by multisystem physiological reserve depletion and homeostatic dysregulation-diminishes a patient's capacity to tolerate the stress of PCI, periprocedural hemodynamic fluctuations, and potential complications (17, 18). This likely represents the primary pathophysiological mechanism underlying the observed increase in post-procedural mortality risk. Notably, the analysis of in-hospital mortality exhibited substantial heterogeneity (*I*^2^ = 98.9%).

An important contribution of this study is the demonstration that frailty is significantly associated with both major bleeding (RR = 2.26, 95% CI: 1.54–3.31) and ischemic stroke (RR = 1.90, 95%CI: 1.86–1.94) in very elderly patients undergoing PCI. This finding highlights the unique “bidirectional risk dilemma” faced by frail patients undergoing PCI and subsequent antithrombotic therapy. On one hand, frailty-related organ dysfunction, coagulation abnormalities, and nutritional decline confer a high bleeding propensity (5), creating a fundamental conflict with the necessity of antithrombotic treatment. On the other hand, the chronic low-grade inflammatory state, endothelial dysfunction, and abnormal platelet reactivity associated with frailty (13) may simultaneously increase the risk of arterial thrombotic events. However, it should be noted that the pooled result for stroke was largely driven by a single large study: after excluding that study, the effect estimate increased (RR = 3.52) but the confidence interval became extremely wide (95% CI: 0.16–77.20) and lost statistical significance (*P* = 0.424), with persistent high heterogeneity (*I*^2^ = 74%). Therefore, although current evidence supports an association between frailty and stroke risk, its robustness is limited and should be interpreted with caution.

In contrast to the hip fracture literature (19), where the focus is predominantly on short-term, in-hospital outcomes, the risk trajectory after PCI is distinguished by its chronic nature, extending over the entire duration of mandatory dual antiplatelet therapy (DAPT). Our pooled analysis of bleeding outcomes demonstrated that frailty significantly increases the risk of major bleeding, an association that remained robust in studies employing standardized bleeding definitions (e.g., BARC type ≥2). Analysis of stroke outcomes suggests that the increased ischemic risk associated with frailty may partially offset the net benefit of antithrombotic therapy, a finding with important clinical implications for guiding antithrombotic strategy selection in very elderly patients.

Considering that the pooled sample included studies with extremely large sample sizes, to determine whether the overall effect estimates were primarily driven by these large datasets, we conducted an influence analysis. The results showed that after sequentially excluding the large studies, the direction of effect for in-hospital mortality and bleeding events remained consistent, indicating that the overall estimates had good robustness.

Our study observed significant heterogeneity across multiple outcome endpoints (In-hospital mortality and Bleeding). Meta-regression analyses were performed for publication year, frailty scale, and myocardial infarction type, and identified myocardial infarction type as a source of heterogeneity. The inherent differences between NSTEMI and STEMI in pathophysiology (incomplete occlusion vs. complete occlusion), treatment urgency, and prognostic determinants lead to different predictive effects of frailty on outcomes, thereby constituting a source of heterogeneity. In subgroup analyses, comprehensive assessment tools (e.g., EFS, CFI) demonstrated the lowest heterogeneity in predicting all-cause mortality and bleeding events (for all-cause mortality, *I*^2^ decreased from 60.1% in the overall analysis to 14.8% in the subgroup using comprehensive tools). These instruments, through their multidimensional assessment framework (encompassing physical function, nutritional status, comorbidity burden, and cognitive function), are able to comprehensively capture the complexity of physiological reserve depletion, thereby providing more robust risk estimates. This finding is consistent with previous conclusions from cardiac surgery research (20), suggesting that comprehensive assessment tools should be the preferred choice in clinical scenarios requiring precise risk stratification. Screening tools [e.g., the FRAIL scale, clinical frailty scale (CFS)] demonstrated moderate heterogeneity in predicting all-cause mortality, yet they offer the distinct advantage of being easy to administer. Notably, screening tools demonstrated a unique advantage in predicting in-hospital mortality (subgroup analysis of studies using screening tools showed heterogeneity *I*^2^ = 29.8%), which may be related to their incorporation of domains such as daily activities, mobility, and energy level. Risk stratification tools, such as the Hospital Frailty Risk Score (HFRS), assess frailty based on electronic health record data. While they offer the advantage of not requiring direct patient contact, their predictive performance is substantially influenced by the completeness and accuracy of coded data, resulting in high heterogeneity in predicting bleeding and stroke outcomes. Study-specific tools, which lack standardized validation, have limited cross-study comparability in their predictive performance, representing an important source of heterogeneity for certain outcomes.

Subgroup analyses revealed that prospective studies demonstrated greater consistency in estimating outcomes such as bleeding, in-hospital mortality, and all-cause mortality. For instance, in-hospital mortality exhibited high heterogeneity in the overall analysis (*I*^2^ = 98.9%), which decreased substantially to 9.8% in the subgroup of prospective studies. This finding highlights the advantages of prospective study design in controlling recall bias, standardizing exposure measurement, and establishing temporal relationships. Retrospective studies, which rely on medical records for frailty ascertainment, may be subject to misclassification bias, which to some degree weakens the strength of the evidence.

Subgroup analysis by geographical region revealed significant regional heterogeneity for most outcomes. As an illustrative example, the pooled relative risk for bleeding events was substantially lower among Asian cohorts (RR = 1.93, 95% CI: 1.29–2.90) compared to their North American counterparts (RR = 2.40, 95% CI: 1.42–4.04). This phenomenon may be related to regional differences in peri-PCI management strategies, DAPT regimen selection, and the intensity of secondary prevention.

Based on this study's systematic comparison of frailty assessment tool categories, we propose the following stratified recommendations: for clinical research scenarios requiring precise risk stratification, comprehensive assessment tools (e.g., EFS, CFI) are recommended as the preferred approach. These tools provide the most robust risk estimates, and although their longer administration time (approximately 15–30 min) is acceptable in research settings, it limits their feasibility in busy catheterization laboratory environments. For rapid screening scenarios in clinical practice, screening tools are recommended as the initial assessment modality. Among these, the Clinical Frailty Scale (CFS) and the FRAIL scale demonstrate high clinical feasibility due to their rapid administration (< 5 min), lack of equipment requirements, and ease of use. The unique advantage of screening tools in predicting in-hospital mortality makes them particularly suitable for emergency decision-making scenarios in patients with acute coronary syndromes. For risk stratification needs at the healthcare system level, risk stratification tools (e.g., HFRS) can serve as a complementary approach for big data-based risk prediction and quality improvement initiatives. However, their predictive performance has certain limitations, and they should not replace direct clinical assessment.

Several limitations of this systematic review and meta-analysis should be acknowledged. First, all included studies were observational in design. The pooled effect estimates we reported were derived from adjusted risk estimates (e.g., multivariable-adjusted odds ratios or risk ratios) as reported by each study. Although we restricted inclusion to cohort studies to minimize bias, potential unmeasured or residual confounding factors (e.g., nutritional status, social support, cognitive function) may still affect causal inference. Moreover, the GRADE quality of evidence was low for all outcomes, so our confidence in the effect estimates is limited, and the true effect may differ from the estimates. Second, the operational definitions and assessment thresholds for “frailty” varied across studies. This was a major source of heterogeneity in our analysis and underscores the urgent need to establish a standardized assessment protocol specifically for very elderly patients undergoing PCI. Third, due to limitations of the primary studies, we were unable to analyze the association between frailty and device-related outcomes such as stent thrombosis or target vessel revascularization, nor were we able to obtain patient-centered data on functional status or quality of life. Fourth, Although the funnel plot was symmetric and the Egger's test and Begg's test did not reveal significant publication bias (*P* = 0.32 for all-cause mortality), the possible absence of unpublished studies with negative results may lead to an overestimation of some effect estimates.

Furthermore, future large-scale, multicenter prospective cohort studies are needed to validate the causal associations between frailty and various outcomes using standardized multidimensional assessment tools. We recommend developing and validating frailty risk assessment models specifically for very elderly PCI populations, integrating clinical, biological, and functional indicators. Future research should explore the interactions between frailty and novel antithrombotic agents (e.g., low-dose rivaroxaban) and interventional techniques (e.g., drug-coated balloons, physiology-guided target lesion revascularization). Importantly, future investigations must integrate patient-reported outcomes-including functional status and health-related quality of life-as core endpoints, thereby advancing the paradigm shift from merely prolonging survival toward preserving meaningful quality of life.

## Conclusion

This systematic review indicates that frailty is an independent risk factor for in-hospital mortality, all-cause mortality, and major bleeding after PCI in very elderly patients aged ≥75 years. Regarding stroke, although the pooled analysis showed that frailty was associated with an increased risk of stroke, this result lost statistical significance after excluding one large study, suggesting limited robustness and warranting cautious interpretation. Subgroup analyses based on the frailty instrument classification framework revealed that comprehensive assessment tools have greater robustness in risk prediction, whereas screening tools, due to their convenience, are suitable for rapid clinical decision-making. Integrating frailty assessment into individualized risk prediction models for older patients with coronary artery disease is of great significance for optimizing treatment decisions and improving patient outcomes. However, the GRADE quality of evidence was low for all outcomes, and caution is needed when generalizing the conclusions.

## Data Availability

The original contributions presented in the study are included in the article/supplementary material, further inquiries can be directed to the corresponding author.
